# Comparative genome analysis revealed gene inversions, boundary expansions and contractions, and gene loss in the *Stemona sessilifolia* (Miq.) Miq. chloroplast genome

**DOI:** 10.1371/journal.pone.0247736

**Published:** 2021-06-18

**Authors:** Jingting Liu, Mei Jiang, Haimei Chen, Yu Liu, Chang Liu, Wuwei Wu

**Affiliations:** 1 Key Laboratory of Bioactive Substances and Resource Utilization of Chinese Herbal Medicine from Ministry of Education, Engineering Research Center of Chinese Medicine Resources from Ministry of Education, Institute of Medicinal Plant Development, Chinese Academy of Medical Sciences, Peking Union Medical College, Beijing, P. R. China; 2 Guangxi Botanical Garden of Medicinal Plants, Nanning, P. R. China; Institute for Biological Research, SERBIA

## Abstract

*Stemona sessilifolia* (Miq.) Miq., commonly known as Baibu, is one of the most popular herbal medicines in Asia. In the Chinese Pharmacopoeia, Baibu has multiple authentic sources and there are many similar herbs sold as Baibu in herbal medicine markets. The existence of counterfeits of Baibu brings challenges to its identification. To assist in its accurate identification, we sequenced and analyzed the complete chloroplast genome of *S*. *sessilifolia* using next-generation sequencing technology. The genome was found to be 154,037 bp in length, possessing a typical quadripartite structure consisting of a pair of inverted repeats (IRs: 27,090 bp) separated by a large single copy (LSC: 81,949 bp) and a small single copy (SSC: 17,908 bp). A total of 112 unique genes were identified, including 80 protein-coding, 28 transfer RNA and four ribosomal RNA genes. In addition, 45 tandem, 27 forward, 23 palindromic and 104 simple sequence repeats were detected in the genome by repeated analysis. Compared with its counterfeits (*Asparagus officinalis* and *Carludovica palmata*) we found that IR expansion and SSC contraction events of *S*. *sessilifolia* resulted in two copies of *the rpl*22 gene in the IR regions and a partial duplication of the *ndh*F gene in the SSC region. An approximately 3-kb-long inversion was also identified in the LSC region, leading to *the pet*A and *cem*A genes being presented in the complementary strand of the chloroplast DNA molecule. Comparative analysis revealed some highly variable regions, including *trn*F*-*GAA_*ndh*J, *atp*B*_rbc*L, *rps*15*_ycf*1, *trn*G*-*UCC*_trn*R*-*UCU, *ndh*F*_rpl*32, *acc*D*_psa*I, *rps*2*_rpoC*2, t*rn*S*-*GCU*_trn*G*-*UCC, *trn*T*-*UGU*_trn*L-UAA and *rps*16*_trn*Q-UUG. Finally, gene loss events were investigated in the context of phylogenetic relationships. In summary, the complete plastome of *S*. *sessilifolia* will provide valuable information for the distinction between Baibu and its counterfeits and assist in elucidating the evolution of *S*. *sessilifolia*.

## Introduction

Radix Stemonae, also known as Baibu, is one of the most popular herbal medicines used in many Asian countries, including China, Korea, Japan, Thailand and Vietnam. It has been used for the treatment of various respiratory diseases such as bronchitis, pertussis and tuberculosis [1, 2]. It was also used for killing cattle parasites, agricultural pests and domestic insects [3, 4]. Stenine B, one of the major chemical ingredients of Baibu, has been considered as a potential drug candidate for use against Alzheimer’s disease due to its significant acetylcholinesterase inhibitory activity [5]. Owing to the important medicinal value, extensive genetic, biochemical and pharmacological studies on Baibu are needed.

According to the Pharmacopoeia of the People’s Republic of China (2015 edition), the root tubers of *Stemona tuberosa*, *S*. *japonica* and *S*. *sessilifolia* were all considered to be authentic sources of Baibu. Although these three species have all been employed as the raw materials of Baibu, we cannot ignore their inherent differences. For example, alkaloids from the genus Stemona are the major components responsible for Baibu’s antitussive activities. However, the chemical composition and content of the different members of the genus *S*. *tuberosa*, *S*. *japonica* and *S*. *sessilifolia* vary greatly [6, 7]. These three species differ in their antitussive, anti-bacterial and insecticidal activities [8]. Therefore, it is critical to determine the exact origin of the plant material used as Baibu.

The existence of multiple authentic sources and the similarities between species increase the difficulty for correctly identifying Baibu. In some areas of China, another herbal medicine, *Aconitum kusnezoffii* Rchb., is also known as Baibu. However, the therapeutic activity of *A*. *kusnezoffii* is significantly different from the authentic sources of Baibu described in the Chinese Pharmacopoeia. Research has even reported that this alternative might result in toxicity when *A*. *kusnezoffii* is taken in larger quantities [9]. In addition, counterfeits in the herbal market also bring challenges to the correct identification of Baibu. Due to their similar morphologic features to the authentic sources of Baibu, many counterfeits such as *Asparagus officinalis*, *A*. *filicinus* and *A*. *acicularis* are often sold as Baibu in the herbal market [10]. Therefore, the distinction between Baibu and its counterfeits is critical for its beneficial usage as a medicinal herb.

Compared to morphological characteristics, a DNA barcode is deemed to be a more efficient and effective method for identifying a particular plant species. Typical barcodes such as *ITS*, *psb*A-*trn*H, *mat*K and *rbc*L have been used to distinguish different plant species [11–13]. However, these DNA barcodes did not always working effectively, especially when trying to distinguish closely related plant species. Such a phenomenon may be attributed to the fact that a single-locus DNA barcode still lacks adequate variations generally observed in closely related taxa. Compared with a general DNA barcode, the chloroplast genome can provide more abundant genetic information and higher resolutions when identifying plant species. Some researchers have proposed using the chloroplast genome as a species-level DNA barcode [14, 15].

The chloroplast is an organelle which is present in almost all green plants. It is central to photosynthesis and plays a vital role in sustaining life on earth by converting solar energy to carbohydrates. Besides photosynthesis, the chloroplast also plays critical roles in other biological processes, including the synthesis of amino acids, nucleotides, fatty acids and many secondary metabolites. Furthermore, metabolites synthesized in chloroplasts are often involved in plants’ interactions with their environment, such as their response to environmental stress and defense against invading pathogens [16–18]. Due to its essential roles in the cellular processes and its relatively small genome size, the chloroplast genome is a good starting point for resolving phylogenetic ambiguity, discriminating closely between related species and revealing the plants’ evolutionary process [19–21]. To date, over 5000 chloroplast genomes from a variety of land plants are available. Phylogenetic analyses have demonstrated the effectiveness of the chloroplast genome in inferring the phylogenetic identity of plants as well as having the ability to distinguish between closely related plant species [22, 23].

Unfortunately, the taxonomic coverage of the sequenced chloroplast genome is somewhat biased. For example, until now, the chloroplast genome of *S*. *sessilifolia* has not been reported. The lack of chloroplast genome information prohibited studies aimed at understanding the evolutionary processes in the family Stemonaceae. Here, we report the full plastid genome of *S*. *sessilifolia*. Based on the sequence data, we performed a multi-scale comparative genome analysis among *S*. *sessilifolia*, *A*. *officinalis* and *Carludovica palmata* (the major counterfeits of Baibu). We investigated the difference among these three species from three aspects, including general characteristics, repeat sequences and sequence divergence. We also characterized the significant changes, including genome rearrangements, IR expansion and SSC contraction, in the plastid genome of *S*. *sessilifolia*, *A*. *officinalis* and *C*. *palmata*.

Lastly, we investigated the gene loss events in Stemonaceae and its closely related families (Asparagoideae, Velloziaceae, Cyclanthaceae and Pandanaceae). The results reported in this work will provide valuable information for species distinction of herb materials that are used as Baibu. Furthermore, it lays the foundation for elucidating the evolutionary history of plant species in the family Stemonaceae.

## Materials and methods

### Plant materials and DNA extraction

Fresh young leaves of *S*. *sessilifolia* from multiple individual plants were collected from the Institute of Medicinal Plant Development (IMPLAD), Beijing, China, and stored at -80°C for chloroplast DNA extraction. All samples were identified by Professor Zhao Zhang, from IMPLAD, Chinese Academy of Medical Sciences & Peking Union Medical College and voucher specimens were deposited in the herbarium of the institute. *S*. *sessilifolia* is not an endangered or protected species and specific permission for the collection of *S*. *sessilifolia* was not required. Total DNA was extracted from 100mg of fresh young leaves using a plant genomic DNA kit (Tiangen Biotech, Beijing, Co., Ltd.). Finally, 1.0% agarose gel and a Nanodrop spectrophotometer 2000 (Thermo Fisher Scientific, United States) were used to evaluate the purity and concentration of the extracted DNA, respectively.

### Genome sequencing, assembly and annotation

According to the standard protocol, the DNA of *Stemona sessilifolia* was sequenced using Illumina Hiseq25000 platform, with insert sizes of 500 bases for the library. A total of 5,660,432 paired-end reads (2 × 250bp) were obtained, and low-quality reads were trimmed with Trimmomatic software [24].

In order to extract reads belonging to the chloroplast genome, we downloaded 1,688 chloroplast genome sequences from the GenBank and constructed a Basic Local Alignment Search Tool (BLASTn) database. All trimmed reads were mapped to this database using the BLASTN program [25], and reads with an E-value > 1E-5 were extracted. The reads were first assembled using the SPAdes software with default parameters [26]. The contigs were then subjected to gap closures using the Seqman module of DNASTAR (V11.0) [27]. Finally, the quality of the assembled genome was evaluated by mapping the reads to the genome using Bowtie2 (v2.0.1) with default settings [28]. For further evaluation, all the barcode sequences of *S*. *sessilifolia* available from the GeneBank were download (S1 File), including *mat*K (1), *pet*D(1), *rbc*L (1), *rpo*C1 (1), *rps*16 (1), *rps*19-*rpl*22-*psb*A (1), *trn*L (3) and *trn*L-*trn*F (2) The numbers enclosed in parentheses represent the number of the barcode sequence. The BLAST program was used to calculate the identity differences between the chloroplast genome sequence of *S*. *sessilifolia* and other barcode sequences. As a result, the barcode, *rps*19*-rpl*22*-psb*A, which is located at the boundary of LSC/IRb, was identifies to have a value of 100%. All the other barcode sequences also gave identity values of 100%, indicating the high reliability of the chloroplast genome sequence.

Gene annotation of *S*. *sessilifolia* chloroplast genome was conducted using CpGAVAS2, which is an integrated plastome sequence annotator and analyzer [29]. The tRNA genes were confirmed with tRNAscan-SE [30] and ARAGORN (V1.2.38) software packages [31]. Then the gene/intron boundaries were inspected and corrected using the Apollo program (V1.11.8) [32]. The Cusp and Compseq programs from EMBOSS (V6.3.1) were used to calculate the GC content [33]. Finally, OrganellarGenomeDRAW [34] was used to generate the circular chloroplast genome map of *S*. *sessilifolia*.

### Repeat sequence analysis

Perl script MISA (http://pgrc.ipk-gatersleben.de/misa/) was used to identify simple sequence repeats (SSRs) with the parameters listed as follows: 74 repeat units for mononucleotide SSRs, 20 repeat units for di- and tri-nucleotide repeat SSRs, and 12 repeat units for tetra-, penta-, and hexanucleotide repeat SSRs. Tandem Repeats Finder was used with parameters of 2 for matches and 7 for mismatches and indels [35]. For the minimum alignment score and the maximum period, size was set to 50 and 500, respectively. Palindrome and forward repeats were identified by the REPuter web service [36]. The minimum repeat size and the similarity cut-off were set to 30 bp and 90%, respectively.

### Comparative genomic analysis

A total of three species, including *S*. *sessilifolia*, *A*. *officinalis* (NC_034777), *Carludovica palmata* (NC_026786), were subjected to multiple sequence alignment using mVISTA with default parameters [37]. Subsequently, 20 introns and 108 intergenic regions shared by *S*. *sessilifolia*, *A*. *officinalis*, and *Carludovica palmata* were extracted using custom MatLab scripts and used to perform sequence divergence analysis. Firstly, the sequences of each intergenic-region/intron were aligned individually using the CLUSTALW2 (v2.0.12) [38] program with options "-type = DNA–gapopen = 10 -gapext = 2". Secondly, pairwise distances were calculated with the Distmat program in EMBOSS (v6.3.1) using the Kimura 2-parameters (K2p) evolution model [39]. We attempted to discover highly divergent regions in order to develop novel molecular markers. To identify the occurrence of genome rearrangement events in the chloroplast genome of *S*. *sessilifolia*, synteny analysis among the three species mentioned above were performed using Mauve Alignment [40].

### Phylogenetic analysis

A total of 11 chloroplast genomes which are distributed into Stemonaceae (3), Cyclanthaceae (1), Pandanaceae (1), Velloziaceae (1) and Asparagoideae (5) were retrieved from the RefSeq database. The protein sequences shared by these chloroplast genomes were used to construct a phylogenetic tree with *Veratrum patulum* and *Paris dunniana* as the outgroup taxa (S1 Table). Fifty-eight proteins were involved, and all these protein sequences were aligned using the CLUSTALW2 (v2.0.12) program with options "-gapopen = 10 -gapext = 2 -output = phylip". Then the Maximum Likelihood (ML) method was adopted to infer the evolutionary history of *S*. *sessilifolia* and the other closely related species. The detailed parameters were "raxmlHPC-PTHREADS-SSE3 -f a -N 1000 -m PROTGAMMACPREV–x 551314260 -p 551314260-o Nicotiana_tabacum, Solanum_lycopersicum -T 20".

## Results

### General characteristics of chloroplast genomes

**Fig 1:**
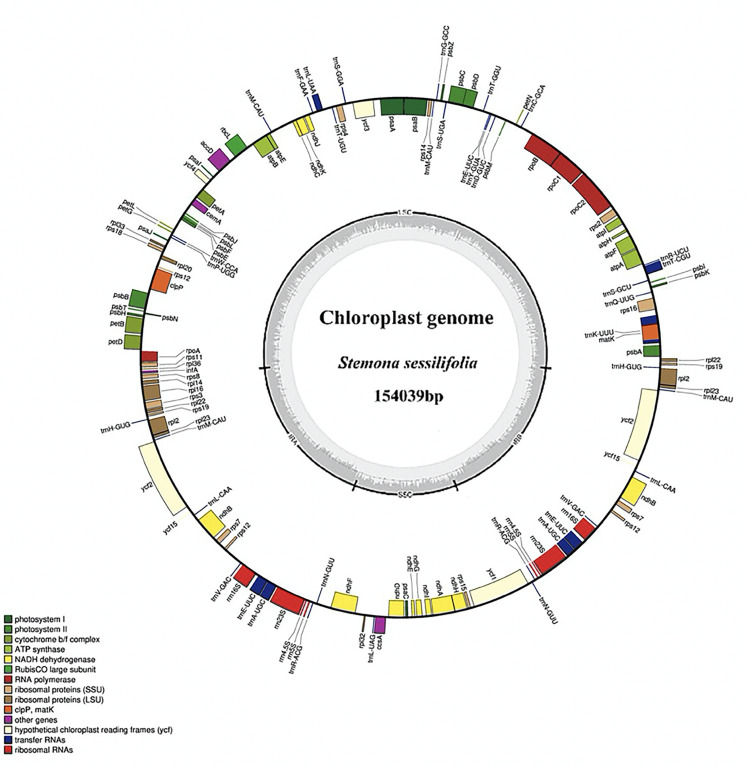
Gene maps of the chloroplast genomes of *Stemona sessilifolia*. Genes inside and outside the circle were transcribed clockwise and counterclockwise, respectively. The darker gray in the inner circle indicates the GC content. Genes with different functions are characterized with different color bars.

The HiSeq2500 generated about 3.2 GB of data and the average coverage depth of the assembled cp genome was 885×. The gene map of *S*. *sessilifolia* is shown in Fig 1. This genome has been deposited in the GenBank (Accession number: MW023922). The chloroplast genomes of *S*. *sessilifolia* and two other species share the standard features of possessing a typical quadripartite structure consisting of a pair of inverted repeats (IRs) separating a large single copy (LSC) and a small single copy (SSC). This is similar to other angiosperm chloroplast genomes [41].

We then carried out a multi-scale comparative genome analysis of these three chloroplast genomes from four aspects, including the size, the guanine-cytosine (GC) content, the number of genes and the gene organization (Table 1). The complete circular chloroplast genomes of *S*. *sessilifolia*, *A*. *officinalis* and *C*. *palmata* were 154,037, 156,699 and 158545 bp, respectively. Compared to *A*. *officinalis* and *C*. *palmata*, *S*. *sessilifolia* showed a relatively short SSC region and a relatively long IR region. We speculated that the chloroplast genome of *S*. *sessilifolia* might undertake IR expansion and SSC contraction simultaneously. There was no significant difference between *S*. *sessilifolia*, *A*. *officinalis*, and *C*. *palmata*. Such a result may be attributed to the high conservation of tRNAs and rRNAs. The lengths of the CDS regions of *A*. *officinalis* and *C*. *palmata* were *found to be* shorter than *S*. *sessilifolia*, indicating that there were probable gene loss events in the chloroplast genome of *A*. *officinalis* and *C*. *palmata*.

**Table 1:** Chloroplast genome characteristics of *Stemona sessilifolia*, *Asparagus officinalis* and *Carludovica palmata*.

Plastome	Characteristics	Species		
		*Stemona sessilifolia*	*Asparagus officinalis*	*Carludovica palmata*	
**Size (bp)**	Genome	154037	156699	158545
	LSC	81949	84999	71426
	IR	27090	26531	26529
	SSC	17908	18638	18364
	tRNA genes	2877	2863	2816
	rRNA genes	9060	9052	8866
	CDS	79260	77436	77802
**GC content (%)**	Overall	38.00	37.59	37.74
	LSC	36.18	35.60	35.79
	IR	42.70	42.92	42.81
	SSC	32.13	31.50	31.51
	tRNA genes	53.43	53.57	53.40
	rRNA genes	55.22	55.38	55.38
	CDS	38.29	38.1	38.41
	1st position	45.7	45.64	45.93
	2nd position	38.46	38.56	38.39
	3rd position	30.72	30.09	30.91
**NO. of genes**	Total	112	110	112
	protein-coding genes	80	78	80
	tRNAs	28	28	28
	rRNAs	4	4	4
	Genes with introns	18	18	18
	Genes in IR	21	22	19

LSC: large single-copy, IR: inverted repeat, SSC: small single-copy, CDS: coding sequence.

As for the GC content, *S*. *sessilifolia* showed a higher value in the regions of the LSC and SSC than *A*. *officinalis* and *C*. *palmate*, even in the complete chloroplast genome. However, in the IR region, *A*. *officinalis* and *C*. *palmata* showed a GC content value larger than *S*. *sessilifolia*. The GC content decreased markedly from the first position to the third position in the codon position scale. Such a result was in line with the phenomenon observed in most land plant plastomes [42–44].

We identified 112, 110 and 112 genes in the chloroplast genomes of *S*. *sessilifolia*, *A*. *officinalis*, and *C*. *palmata*, respectively. All of these three chloroplast genomes have 28 tRNAs and four rRNAs. The number of genes with introns in each species is 18, similar to reports in prior works [45]. Therefore, we may conclude that there were no intron loss events that occurred in the chloroplast genomes of these three species. Among these 18 genes, 16 of them (10 protein-coding genes and 6 tRNAs) had one intron each, 2 genes (*ycf*3 and *clp*P) have two introns each and all of the genes with introns are described in S2 Table. The *rps*12 gene was divided into 5’-*rps*12 in the LSC region and 3’-*rps*12 in IR region. In addition, 20, 22 and 19 genes were predicted for *S*. *sessilifolia*, *A*. *officinalis*, and *C*. *palmata* in the IR regions, respectively.

The gene organization of the three species were compared and the results are presented in Table 2. In the upstream and downstream regions of the *A*. *officinalis* chloroplast genome, premature stop codons were discovered in the *ycf*1 gene, resulting in the loss of this gene. Compared to *S*. *sessilifolia*, we found the shorter CDS regions of *A*. *officinalis* was directly related to the loss of this gene. We also found a full-length and a pseudogene of the *ndh*F gene which coexists in the chloroplast genome of *S*. *sessilifolia*, which indicated the presence of another SSC contraction event.

**Table 2:** Genes presented in chloroplast genomes of *Stemona sessilifolia*, *Asparagus officinalis* and *Carludovica palmata*.

Category for genes	Group of genes	Name of genes
Ribosome RNA genes	rRNA genes	*rrn*16S^a^, *rrn*23S^a^, *rrn*5S^a^, *rrn*4.5S^a^
Transfer RNA genes	tRNA genes	*trn*T*-*UGU, *trn*R-ACG^a^, *trn*T-GGU, *trn*S-UGA, *trnf*M-CAU, *trn*F-GAA, *trn*L-UAG, *trn*V-UAC*, *trn*L-CAA^a^, *trn*M-CAU, *trn*G-GCC, *trn*Q-UUG, *trn*A-UGC^a^, **, *trn*D-GUC, *trn*P-UGG, *trn*I-CAU^a^, *trn*E-UUC**, *trn*L-UAA**, *trn*K-UUU**, *trn*W-CCA, *trn*Y-GUA, *trn*I-GAU^a,^*, *trn*G-UCC*, *trn*S-GGA, *trn*R-UCU, *trn*H-GUG^a^, *trn*S-GCU, *trn*N-GUU^a^, *trn*V-GAC^a^, *trn*C-GCA
Others	Large subunit of ribosome	*rpl*14, *rpl*16*, *rpl*2^a,^*, *rpl*20, *rpl*22^a^, *rpl*23^a^, *rpl*32, *rpl*33, *rpl*36
	Small subunit of ribosome	*rps*11, *rps*12^a,b,^*, *rps*14, *rps*15, *rps*16*, *rps*18, *rps*19^a^, *rps*2, *rps*3, *rps*4, *rps*7^a^, *rps*8
	DNA dependent RNA polymerase	*rpo*A, *rpo*B, *rpo*C1*, *rpo*C2
	Subunits of NADH dehydrogenase	*ndh*A*, *ndh*B^a,^*, *ndh*C, *ndh*D, *ndh*E, *ndh*F, *ndh*G, *ndh*H, *ndh*I, *ndh*J, *ndh*K
	Subunits of cytochrome b/f complex	*pet*A, *pet*B*, *pet*D*, *pet*G, *pet*L, *pet*N
	Subunits of photosystem I	*psa*A, *psa*B, *psa*C, *psa*I, *psa*J
	Subunits of photosystem II	*psb*A, *psb*B, *psb*C, *psb*D, *psb*E, *psb*F, *psb*I, *psb*J, *psb*K, *psb*L, *psb*M, *psb*N, *psb*T, *psb*Z, *ycf*3
	Large subunit of rubisco	*rbc*L
	Subunits of ATP synthase	*atp*A, *atp*B, *atp*E, *atp*F*, *atp*H, *atp*I
	Subunit of Acetyl-CoA-carboxylase	*acc*D
	C-type cytochrome synthesis gene	*ccs*A
	Envelope membrane protein	*cem*A
	Protease	*clp*P**
	Translational initiation factor	*inf*A
	Maturase	*mat*K
	Conserved open reading frames	*ycf*1, *ycf*2^a^, *ycf*15**, *ycf*4
	Pseudogenes	*ycf*1^ψ^, *ndh*F^ψ^, *infA*^ψ^, ***ycf*15** ^a,ψ^, *ycf*68 ^a,ψ^

*Gene with one intron

**Gene with two introns, a Gene with two copies, b Trans-splicing gene, ψ Pseudo gene. **Genes in Bold font** were only identified in *S*. *sessilifolia* and *A*. *officinalis*.Genes with underline were only identified in *A*. *officinalis*.

### Repeat sequence analysis

**Fig 2:**
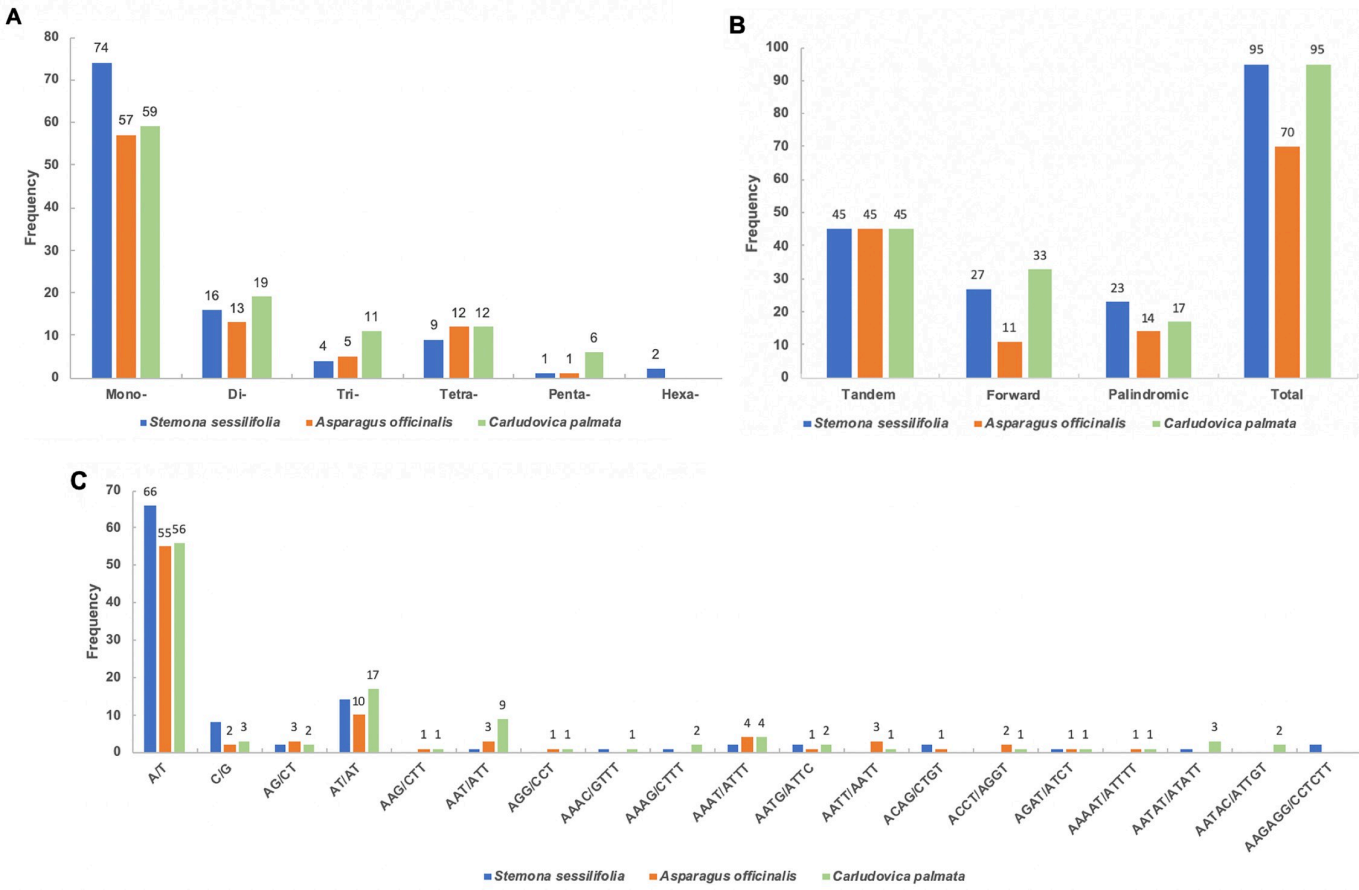
Simple sequence repeats (SSRs) and long repeat sequences identified in the chloroplast genomes. (A) Distribution of different types of SSRs in the chloroplast genomes. (B) Distribution of long repeat sequences in the chloroplast genomes. (C) Frequency of SSR motifs in different repeat class types.

Simple sequence repeats (SSRs), which are tandem repeat sequences consisting of 1–6 repeat nucleotide units, are widely distributed in prokaryotic and eukaryotic genomes. A high degree of polymorphisms of SSRs has been considered to be effective molecular markers when considering species identification, population genetics and phylogenetic research [46, 47]. In the current study, we investigated the distribution of SSRs in the genomes as well as their numbers and types (Fig 2). As a result, a total of 106, 88 and 107 SSRs were detected in *S*. *sessilifolia*, *A*. *officinalis* and *C*. *palmata*, respectively. Mononucleotide motifs showed the highest frequency of SSRs in these species, followed by dinucleotides, tetranucleotides, trinucleotides and pentanucleotides, respectively. However, hexanucleotide repeats were also detected only in *S*. *sessilifolia*. As expected, the majority of repeats consisted of A/T and AT/AT repeats which suggest that these chloroplast genomes are rich in short poly-A and poly-T motifs, while poly-C and poly-G ones are relatively rare. These SSRs were highly polymorphic, suggesting they present great potential for the identification of these three species. We then use Tandem Repeats Finder [35] and REPuter [36] to detect long repeats and found 95, 70, and 95 long repeat sequences in *S*. *sessilifolia*, *A*. *officinalis* and *C*. *palmata*, respectively. Tandem, forward and palindromic repeats were present in all these three species with the number of tandem repeats being the same in all of them. In comparison, the number of forward and palindromic repeats were different in the three species. These two types of repeats were most common in *S*. *sessilifolia* (27 (54%) and 23 (46%), respectively) and least common in *A*. *officinalis* (11 (44%) and 14 (56%), respectively).

In summary, there are significant differences in the types of repeat sequences among *S*. *sessilifolia*, *A*. *officinalis*, and *C*. *palmat*a. The occurrence of repeat events in *S*. *sessilifolia* was higher than that of *A*. *officinalis* and *C*. *palmat*a. It should be noted that the size of *the A*. *officinalis* and *C*. *palmata* chloroplast genome is larger than the chloroplast genome of *S*. *sessilifolia*. Hence, the relatively larger sizes of the chloroplast genomes of *A*. *officinalis* and *C*. *palmata* do not result in many repeat sequences.

### Sequence divergence analysis

**Fig 3:**
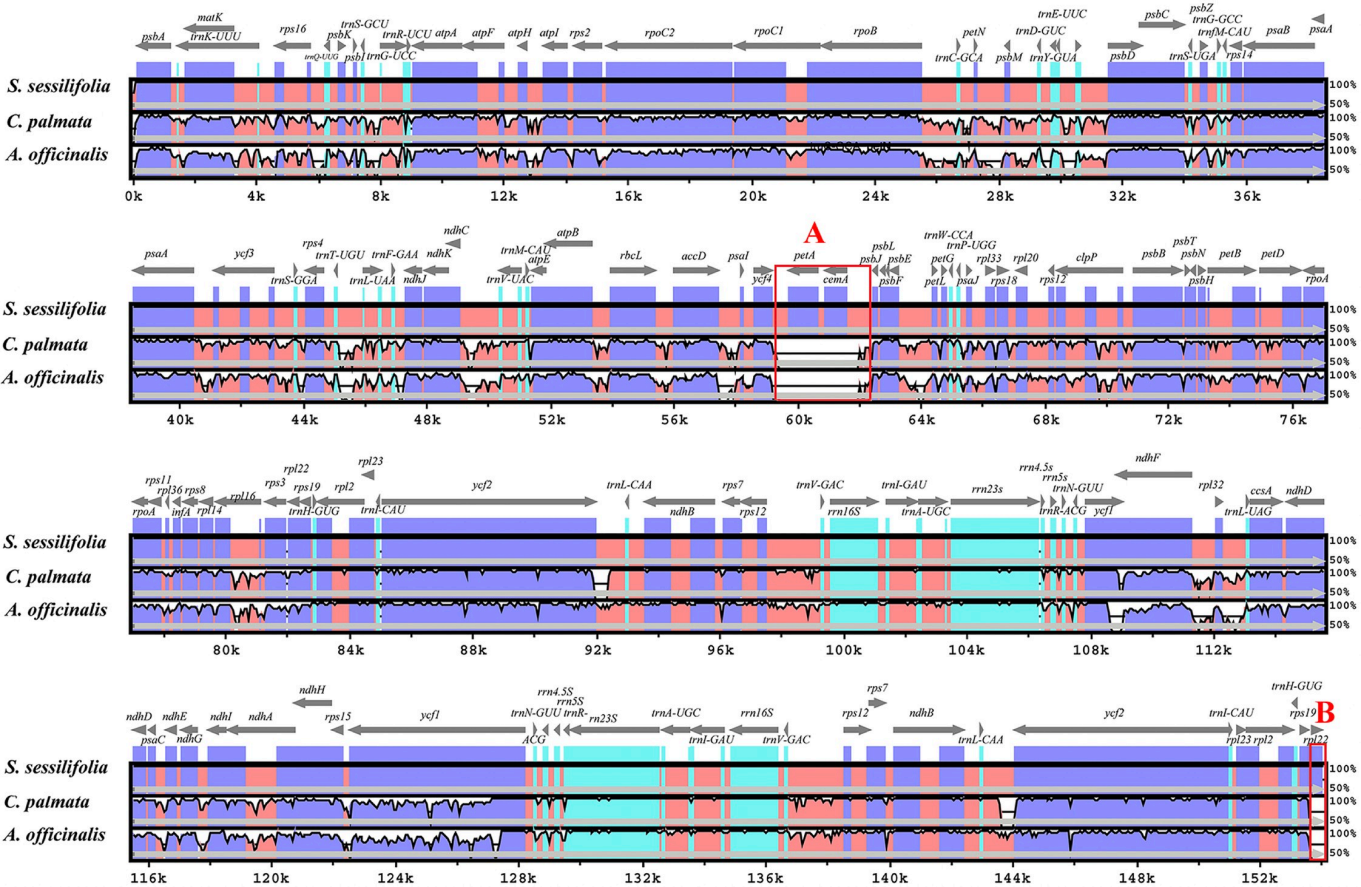
Comparison of the three chloroplast genomes using mVISTA program. The gray arrows indicate the orientations and positions of genes. Untranslated, conserved non-coding and coding regions were characterized by sky-blue, red and blue blocks, respectively. A cut-off value of 70% was adopted during the process of alignment.

To evaluate the genome sequence divergence, we aligned the sequences from three species using mVISTA [37] (Fig 3). The chloroplast genome of *S*. *sessilifolia* was found to be significantly different from *A*. *officinalis* and *C*. *palmata*. As mycoheterotrophic plants, severe gene loss events always lead to highly reduced plastomes [23, 48]. As expected, the non-coding regions were more divergent than coding regions among these species. The two most divergent regions were the *ycf*4-*psb*J region (red square A) and the *rpl*22 coding region (red square B). We suspected that such a phenomenon might be attributed to gene loss events or genome rearrangement events, and this will be discussed in detailed later. The *Ycf*1 gene is also highly divergent, which may be due to the occurrence of pseudogenization. In summary, the LSC region showed the highest divergence, followed by the SSC region and the IR region was less divergent than the LSC and SSC regions. Compared to the coding areas, the intergenic spacers displayed higher divergence areas.

Highly divergent regions can usually assist in the development of molecular markers. Based on the fact that non-coding regions usually evolved more rapidly than coding regions, the intergenic and intron regions are always considered to be ideal candidate regions for molecular markers with high resolution. Therefore, we calculated the Kimura 2-parameter (K2p) distances for each set of the intergenic and intron regions. A relatively higher K2p value between any two species is necessary to distinguish each species from any other two species. Therefore, we calculated the minimal K2p (MK2p) value for each set of intergenic and intron regions. The non-coding regions with higher MK2p values are likely to be the candidate regions for high-resolution molecular markers. Consequently, for introns (S3 Table), the MK2p value ranges from 0.0055 to 0.1096. *Clp*P_intron2 with the highest MK2p value was followed by *rpl*16_intron1. The third, fourth and fifth were *rps*16_intron1, *ndh*A_intron1 and *trn*L-UAA_intron1, respectively. For intergenic spacers (S4 Table), five highly conserved intergenic spacers were observed and these were *ndh*A_*ndh*H, *psa*B_*psa*A, *psb*L_*psb*F, *rpl*2_*rpl*23 and *trn*I-GAU_*trn*A-UGC. The MK2p value of intergenic spacers ranges from 0 to 0.3301, and the top-10 intergenic spacers with higher MK2p values are listed as follows: *trn*F-GAA_*ndh*J, *atp*B*_rbc*L, *rps*15*_ycf*1, *trn*G-UCC*_trn*R-UCU, *ndh*F*_rpl*32, *acc*D*_psa*I, *rps*2*_rpo*C2, t*rn*S-GCU*_trn*G-UCC, *trn*T-UGU*_trn*L-UAA and *rps*16*_trn*Q-UUG. In conclusion, compared to introns, we observed higher sequence divergence in intergenic spacers. The intergenic spacers with large K2p values represent good candidate molecular markers for distinguishing these three species.

### Rearrangement of the chloroplast genome

**Fig 4:**
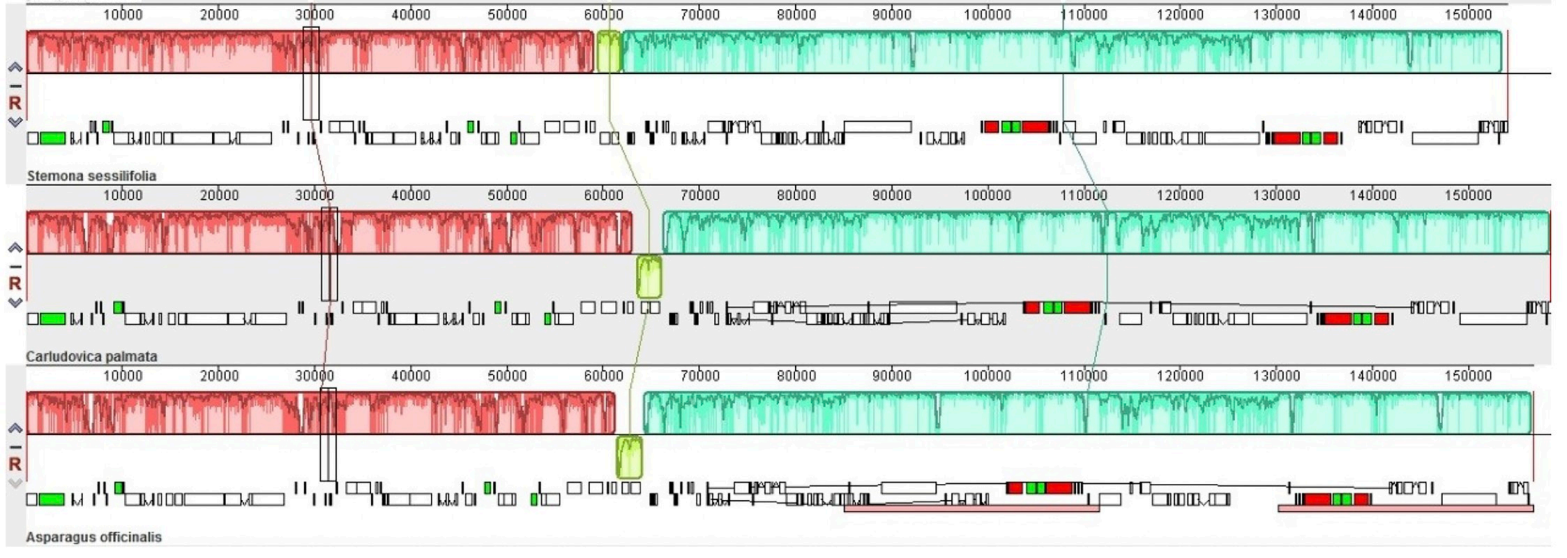
Comparison of the three chloroplast genomes using the MAUVE algorithm. Local collinear blocks were colored to indicate syntenic regions, and the histograms within each block indicated the degree of sequence similarity.

To investigate whether there are significant differences in the *yc*4-*psb*J regions (red square A in Fig 3) and *rpl*22 coding regions (red square B in Fig 3) between *S*. *sessilifolia* and its major counterfeits, we conducted synteny analysis. As plotted in Fig 4, we detected a large inversion of 3 kb long in the LSC region. Interestingly, a similar sequence of an approximately 3-kb long inversion was confirmed to be located in the *ycf*4-*psb*J regions. Therefore, we can conclude that the occurrence of genome rearrangement events leads to a significant difference in *the ycf*4*-psb*J areas between *S*. *sessilifolia* and the other two species. To investigate whether the existence of such an inversion in *S*. *sessilifolia* is unique occurrence, we conducted synteny analysis between the chloroplast genome of *S*. *sessilifolia* and species in Dioscoreales and Liliales, which belong to two closely related orders of Pandanales. Compared to any of the species in Dioscoreales and Liliales, the inversion in the *ycf*4*-psb*J region in *S*. *sessilifolia* was always visible (data not shown). Therefore, the inversion in the *ycf*4*-psb*J areas is probably unique to *S*. *sessilifolia*.

### IR expansion and SSC contraction

**Fig 5:**
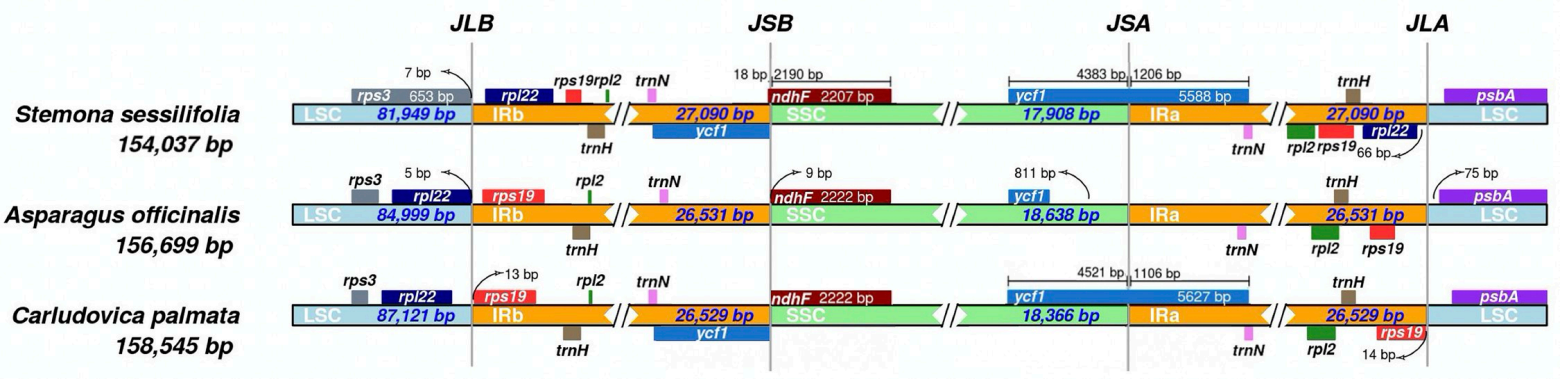
Comparison of IR, LSC and SSC regions among *Stemona sessilifolia*, *Carludovica palmata* and *Asparagus officinalis*. The numbers above, below or adjacent to genes represent the length of genes or the distances from the front or end of genes to the boundary sites. It should be pointed out that the figure features are not to scale.

IR contractions and expansions are common evolutionary events contributing to chloroplast genomes size variation [49]. Here, the JL (LSC/IR) and JS (IR/SSC) boundary comparison analysis was performed by which we attempted to identify IR contraction and expansion events (Fig 5). Compared to *A*. *officinalis* and *C*. *palmata*, the relatively larger IR regions indicated the occurrence of IR expansion events in *S*. *sessilifolia*. Simultaneously, the SSC region was shorter than *A*. *officinalis* and *C*. *palmate* by 465-737bp, suggesting the occurrence of SSC contraction events in *S*. *sessilifolia*. For *A*. *officinalis* and *C*. *palmata*, one copy *of* the *rpl*22 gene is located at the LSC region. However, the IR regions of *S*. *sessilifolia* span to the intergenic spacers between *the rpl*22 and *rps*3 genes, resulting in the presence of two copies of the *rpl*22 gene. Therefore, we can claim that the significant differences in *rpl*22 coding regions between *S*. *sessilifolia* and its major counterfeits can be attributed to the occurrence of IR expansion events.

Furthermore, the *ndhF* gene located at SSC regions in *A*. *officinalis* and *C*. *palmata*, ranges from 9-40bp away from the SSC/IRb junction. However, in *S*. *sessilifolia*, the shortening of the SSC region leads to the *ndh*F gene extending into the IRb region by 18bp. The occurrence of *the ndh*F gene located at the SSC/IRb junction resulted in partial duplication of this gene at the corresponding region. The *ycf*1 gene is found at the IRb/SSC junction, creating a *ycf*1 pseudogene in *S*. *sessilifolia* and *C*. *palmata*. Considering that premature stop codons were discovered in the *ycf*1 gene, only one *ycf*1 gene was annotated in the SSC region in *A*. *officinalis*. An overlap of 18bp between the *ndh*F gene and the *ycf*1 pseudogene was also observed in *S*. *sessilifolia*. In summary, compared to *A*. *officinalis* and *C*. *palmata*, significant junction expansion and contraction events were observed in *S*. *sessilifolia* simultaneously, which were probably responsible for the length variations of these three cp genomes sequences.

### Phylogenetic analysis

**Fig 6:**
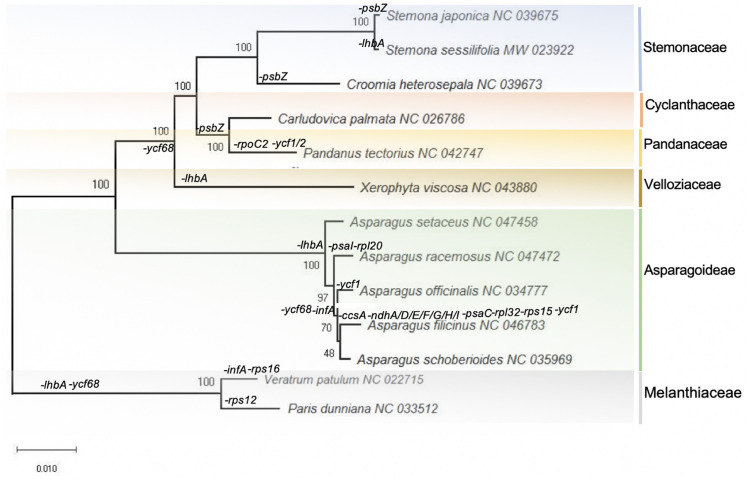
Molecular phylogenetic analysis of Pandanales and its closely related orders. The tree was constructed with 58 protein sequences presented in 116 species using the Maximum Likelihood method and implemented in RAxML with *Nicotiana tabacum* and *Solanum lycopersicum* serving as the outgroups. The numbers associated with the nodes indicate bootstrap values tested with 1000 replicates. The orders and families to which each species belongs are marked beside the branches as well as the occurrence of gene loss events.

The chloroplast genome has been successfully used to determine plant categories and reveal plant phylogenetic relationships [50, 51]. To determine the phylogenetic position of *S*. *sessilifolia*, we constructed a phylogenetic tree with species in Stemonaceae and its closely related families (Asparagoideae, Velloziaceae, Cyclanthaceae and Pandanaceae). A total of 13 chloroplast genomes were retrieved from the RefSeq database, and 58 protein sequences shared by these species were used to construct a phylogenetic tree with *Veratrum patulum* and *Paris dunniana* serving as the outgroups (Fig 6). As a result, the species in Stemonaceae, Asparagoideae and Velloziaceae formed a cluster, respectively. In addition, *S*. *sessilifolia* and *S*. *japonica* formed a cluster within Stemonaceae with a bootstrap value of 100%, indicating a sister relationship between these two species.

As showed in Fig 6, a series of gene loss events were observed throughout Stemonaceae and its closely related families (Asparagoideae, Velloziaceae, Cyclanthaceae and Pandanaceae). A total of 21 genes are lost in these species, including *ycf*68 (11), *lhb*A (9), *inf*A (4), *psb*Z (4), *ycf*1 (3), *ccs*A (1), *ndh*A (1), *ndh*D (1), *ndh*E (1), *ndh*F (1), *ndh*G (1), *ndh*H (1), *ndh*I (1), *psa*C (1), *psa*I (1), *ycf*2 (1), *rps*16 (1), *rpl*20 (1), *rpo*C2 (1), *rps*12 (1) and *rps*15 (1). The numbers enclosed in parentheses represent the frequency of gene loss events. As expected, closely related species always tend to undergo the same gene loss events. A series of clusters formed by species which undergo the same gene deletion events further confirmed such a phenomenon. *C*. *palmata* and *P*. *tectorius* formed a cluster, and both of these species lack the *psb*Z gene. The species from Pandanales (Steminaceae, Cyclanthaceae, Pandanaceae and Velloziaceae) formed a cluster without the ycf68 gene. The species from Asparagoideae formed a cluster without the *lhb*A gene.

The *Ycf*68 gene has the highest frequency of gene deletions, and the second was the *lhb*A gene. The following three genes were *inf*A, *psb*Z and *ycf*1, respectively. Actually, the *ycf*68 gene was only found in two species (*A*. *racemosus* and *A*. *setaceus*), and *the lhb*A gene was only found in four species (*C*. *palmata*, *C*. *heterosepala*, *P*. *tectorius* and *S*. *japonica*). The functions of the *ycf*68, *lhb*A and *ycf*1 genes remain unknown. The occurrence of premature stop codons may account for the rare existence of these three genes in chloroplast genomes [41, 52, 53]. As one of the most active genes in the chloroplast genome, *the inf*A gene plays an essential role in protein synthesis. The frequent absence of the *inf*A gene may be attributed to the transfer of this gene between the cytoplasm and nucleus [41, 54]. The absence of the subunits of the photosystem II gene, *psb*Z, was frequently observed in Pandanales (Steminaceae, Cyclanthaceae and Pandanaceae). For each of the remaining 16 genes, only one gene loss event was observed, respectively. There was a variety of gene absences in the chloroplast genome of each species, indicating the diversity of variations in the chloroplast genomes. However, for 16 out of 21 genes, the frequency of gene loss events was limited to only one, suggesting that the chloroplast genome is highly conserved at the scale of gene content. Such a phenomenon is consistent with the highly conserved nature of the chloroplast genome as well as its feature of rich variations.

## Discussion

Chloroplast genome is frequently used for species identification and plant phylogenetics at generic level [42, 50]. It has also been used at the family level to infer family level phylogenetic relationships and species identification events [55]. Counterfeits medicine are a type of crude drug preparation with a similar morphology but having lower effective components when compared to the authentic medicine. Therefore, it is an important task to distinguish traditional Chinese medicine and its counterfeits. In this study, we sequenced and analyzed the chloroplast genome of *S*. *sessilifolia* and performed multi-scale comparative genomics of *S*. *sessilifolia* and the major counterfeits of Baibu, *A*. *officinalis*, and *C*. *palmata*. We also characterized the major changes in the chloroplast genome of these three species, including genome rearrangements, IR expansions and SSC contractions, and investigated the occurrence of gene loss events in Dioscoreales, Liliales, Pandanales and Asparagaceae. Such chloroplast genome analyses can broaden the knowledge regarding the genome organization and phylogenetics of *S*. *sessilifolia* and its counterfeits. In addition, two divergence hotspots and 10 intergenic spacers with large K2p values were found in the current study and these might be used for the development of molecular markers.

Our results show that the genome organization and content as well as the synteny characteristics were similar among *S*. *sessilifolia*, *A*. *officinalis*, and *C*. *palmata*. This could be attributed to the fact that chloroplast genomes of land plants have conserved features [56–58]. Nevertheless, previous studies have shown that different regions of the chloroplast genome have different GC content [56, 58], while the IR region has high GC content due to the existence of rRNAs which have high GC content. These three species also have similarities in genes content and genome organization. Interestingly, a large inversion was found in *S*. *sessilifolia*. The reverse orientation of the SSC region has also been reported in a wide variety of plant species, such as *S*. *japonica*, *Croomia heterosepala* and *C*. *japonica*, which all belong to Stemonaceae [59]. By constrast, *A*. *officinalis* and other species such as *Salvia miltiorrhiza* [60] and Cornales [61] do not have this inversion of the SSC region. This phenomenon is sometimes interpreted as a major inversion existing within the species [62–64]. In fact, the two orientations of the SSC region have been found to occur regularly during the course of chloroplast DNA replication within individual plant cells [65, 66]. Therefore, the reverse orientation of the SSC region in *S*. *sessilifolia* and other Stemona species may represent a form of chloroplast heteroplasmy.

SSRs have been widely used as molecular markers in studies of species identification, population genetics and phylogenetic investigations based on their high-degree of variations [67]. The SSRs consisting of mononucleotide A/Ts are the most abundant types in *S*. *sessilifolia*, *A*. *officinalis* and *C*. *palmata*. A similar trend of SSRs was also reported in the chloroplast genomes of not only Stemona species but also in other families of angiosperms [56, 59, 68]. These SSRs sequences are often composed of simple repeating units such as polyadenine (PolyA) or polythymine (Poly T) repeats, which have a significant impact on the overall G/C content of the genomes [69]. With the length of polymorphisms in *S*. *sessilifolia*, *A*. *officinalis* and *C*. *palmata*, they suggest great potential for use in the identification of these three species.

Previous studies have shown that highly divergent regions identified by comparative genomics can reveal sites that can be used for DNA barcoding [68, 70]. Such divergent sites in the chloroplast can be applied to DNA barcoding [43, 44, 71–73]. Here, we determined a 3-kb long inversion in the chloroplast genome of *S*. *sessilifolia* which might result from a genome rearrangement event. This unique inversion phenomenon led to significant differences in the *ycf*4*-psb*J region among *S*. *sessilifolia*, *A*. *officinalis* and *C*. *palmata*, which can be used as a candidate region to identify *S*. *sessilifolia* from counterfeits. Furthermore, the 10 intergenic spacers with large MK2p values in our study could be applied to DNA barcoding. Fan *et al*. reported the nucleotide sequences of chloroplast DNA *trn*L-*trn*F, *trn*H-*psb*A, *pet*B-*pet*D and *trn*K-*rps*16 regions which can provide useful information in order to discriminate the Stemona species (*S*. *sessilifolia*, *S*. *japonica* and *S*. *tuberosa*), as well as the common counterfeits such as the *Asparagus* species [10]. Among them, nucleotide variations were found in the partial sequences of the *rps*16 and *trn*L genes. In our study, *trn*T-UGU*_trn*L-UAA and *rps*16*_trn*Q-UUG were also among the top 10 intergenic spacers with higher MK2p values. Whether there are variable sites in the intergenic regions that we found lead to large MK2p values still needs to be further elucidated. Lu *et al*. compared the pairwise sequence divergence values across all introns and intergenic spacers in two Stemona species (*S*. *japonica* and *S*. *mairei*) revealed that the *ndh*F–*rpl*32 and *trn*S–*trn*G regions were the fastest-evolving regions. These findings agreed with our results. These regions are therefore likely to be the choices for molecular evolutionary and systematic studies between *S*. *sessilifolia* and its counterfeits.

In addition, there were significant differences in the IR contractions and expansions between *S*. *sessilifolia* and the other two species. As far as the JLB (IRb/LSC) boundary is concerned, we found that *S*. *sessilifolia* was significantly expanded in the IR region, which led to the presence of two copies of the *rpl*22 gene. Besides, SSC region contraction resulted in partial duplication of the *ndh*F gene at the corresponding region and the *ycf*1 pseudogene in *S*. *sessilifolia*. It was reported that the *ndh*F gene is involved in photosynthesis, and it was often detected during the positive selection that occurs during the evolutionary process of the species [44, 74]. The function of the *ycf*1 genes is mostly unknown, but it is known to evolve rapidly [21]. The contraction and expansion of the IR region in *S*. *sessilifolia* suggests that there is a significant difference in gene sequences between *S*. *sessilifolia* and its counterfeits, and it is important to understand the genome structure and evolutionary process of the chloroplast genome.

Phylogenetic analyses showed that *S*. *sessilifolia* and *S*. *japonica* (both of which are authentic sources of Baibu according to Pharmacopoeia of the People’s Republic of China (2015 edition) were placed close to each other with bootstrap values of 100%, while *A*. *officinalis* and *C*. *palmata* were on the other branches. When we investigated the gene loss events in the context of phylogenetic relationships, we also found that the cp genomes of *S*. *sessilifolia* and *S*. *japonica* have similar gene loss patterns. These findings support the pharmaceutical use of *S*. *sessilifolia* and *S*. *japonica as* genuine Baibu, and also suggest the urgent need for finding new molecular markers for the identification of genuine Baibu. This study will be of value in determining genome evolution and understanding the phylogenetic relationships within Pandanales and other closely related species.

## Conclusions

In summary, the complete plastome of *S*. *sessilifolia* (Miq.) Miq. is provided in the current study. We believe it will be of benefit as a reference for further complete chloroplast genome sequencing within the family. Based on sequence data provided, a multi-scale comparative genome analysis of *S*. *sessilifolia and* the major counterfeits of Baibu, *A*. *officinalis* and *C*. *palmata*, was performed. Comparative analysis of these three species revealed the existence of a unique inversion in the *ycf*4*-psb*J regions. Interestingly, IR expansion and SSC contraction were observed in *S*. *sessilifolia* simultaneously, resulting in a rare boundary pattern. Some highly variable regions were screened as potential DNA barcodes for identification of these three species, including *tr*nF-GAA_*ndh*J, *atp*B*_rbc*L, *rps*15*_ycf*1, *trn*G-UCC*_trn*R-UCU, *ndh*F*_rpl*32, *acc*D*_psa*I, *rps*2*_rpo*C2, t*rn*S*-*GCU*_trn*G-UCC, *trn*T-UGU*_trn*L-UAA and *rps*16*_trn*Q-UUG. Phylogenetic analyses showed that the two Stemona species were placed close to each other with a bootstrap value of 100%. Finally, we investigated the gene loss events in the context of the phylogenetic relationship. It is obvious that closely related species always tend to share similar gene loss patterns, consistent with those observed previously. This study will be of value in determining genome structure differences, which can be utilized to identify *S*. *sessilifolia* and its counterfeits and understanding the phylogenetic relationships within Stemonaceae and its closely related families.

## Supporting information

S1 File The barcode sequences of *Stemona sessilifolia* available in GeneBank. (FASTA)

S1 Table List of chloroplast genomes used in this study. (XLSX)

S2 Table The length of introns and exons for intron-containing genes. (DOCX)

S3 Table K2p distances of the intron regions in *Stemona sessilifolia*, *Carludovica palmata* and *Asparagus officinalis*. (XLSX)

S4 Table K2p distances of the intergenic regions in *Stemona sessilifolia*, *Carludovica palmata* and *Asparagus officinalis*. (XLSX)
