# Supplementary material for: Comparative genome analysis revealed gene inversions, boundary expansions and contractions, and gene loss in the Stemona sessilifolia (Miq.) Miq. chloroplast genome
Source: PLoS One. 2021 Jun 18;16(6):e0247736. doi: 10.1371/journal.pone.0247736 (PMC8213164; doi:10.1371/journal.pone.0247736)
Supplement: S2 Table — (DOCX) [file pone.0247736.s003.docx]

**S2 Table. The length of introns and exons for intron-containing genes.**

| **Gene** | **Exon I** | **Intron I** | **Exon II** | **Intron II** | **Exon III** |
| --- | --- | --- | --- | --- | --- |
| *trnK-UUU* | 37 | 2575 | 35 |  |  |
| *rps16* | 40 | 854 | 212 |  |  |
| *trnG-UCC* | 23 | 730 | 48 |  |  |
| *atpF* | 145 | 774 | 410 |  |  |
| *rpoC1* | 432 | 690 | 1608 |  |  |
| *ycf3* | 131 | 724 | 229 | 733 | 159 |
| *trnL-UAA* | 35 | 508 | 50 |  |  |
| *trnV-GAC* | 39 | 583 | 37 |  |  |
| *clpP* | 71 | 782 | 291 | 666 | 250 |
| *petB* | 6 | 791 | 642 |  |  |
| *petD* | 6 | 769 | 516 |  |  |
| *rpl16* | 9 | 1020 | 402 |  |  |
| *rpl2* | 409 | 660 | 428 |  |  |
| *ndhB* | 775 | 700 | 758 |  |  |
| *rps12* | 114 |  | 232 | 550 | 26 |
| *trnI-GAU* | 42 | 940 | 35 |  |  |
| *trnA-UGC* | 37 | 802 | 36 |  |  |
| *ndhA* | 544 | 1046 | 530 |  |  |

The rps12 gene is divided into 5'-rps12 in the LSC region and 3'-rps12 in IR regions.
